# Enhancement of Dough Processing and Steamed Bread Quality with Modified Soybean Residue Dietary Fiber

**DOI:** 10.3390/foods14030346

**Published:** 2025-01-21

**Authors:** Jun Zhao, Wenlong Xie, Zhilong Chen, Yuqian Zheng, Sheng Li

**Affiliations:** College of Food Science and Engineering, Changchun University, Changchun 130022, China; zhaoj70@ccu.edu.cn (J.Z.); xwl875294619@163.com (W.X.); 13843949119@163.com (Z.C.); zhengyuqian0226@163.com (Y.Z.)

**Keywords:** modified soybean residue dietary fiber, ultrasound combined with enzyme method, wheat dough, steamed bread, sensory attributes

## Abstract

The effects of different modified soybean residues’ dietary fiber on the physicochemical properties of wheat dough and the quality of steamed bread were systematically analyzed in this study. The physical and chemical parameters of dough, such as texture characteristics, water distribution, secondary structure, and the specific volume, color, and sensory evaluation results of steamed bread products were analyzed in detail. The results showed that adding 6% modified soybean residue dietary fiber enhanced the gluten network, increasing the S–S bond content and improving gluten stability. Notably, the inclusion of 6% residue modified by the ultrasound combined with enzyme method (UEDF) led to a 2.55% increase in the β-fold content of gluten proteins and a 3.60% rise in disulfide bond content. These changes resulted in a reduction in dough relaxation time, promoting a more uniform and compact pore structure in the dough. Additionally, steamed bread made with 6% UEDF showed a 0.3 mL/g increase in specific volume, a 4.69 point rise in L* value, and improved sensory attributes such as taste, odor, and appearance. These research results provide valuable insights and guidance for the development of soybean residue dietary fiber foods.

## 1. Introduction

Steamed bread is a traditional Chinese food made from wheat flour through a process of mixing, fermenting, and steaming. It is valued for its simplicity and nutritional benefits. However, one drawback is that consuming steamed bread can lead to a rapid increase in blood sugar levels [1]. In response to growing consumer demand for stable blood sugar levels, more manufacturers are turning to high-fiber foods. Dietary fiber is widely recognized for its potential to lower blood sugar and reduce low-density lipoprotein (LDL) levels, which can help prevent cardiovascular diseases.

Soybean residues, a byproduct of soybean processing, are rich in nutrients such as dietary fiber, protein, fat, and minerals [2]. However, the coarse texture of soybean residue dietary fiber can make it challenging to incorporate directly into steamed bread, as it may result in a gritty texture that impacts the eating experience. Additionally, the inclusion of dietary fiber can slow down fermentation, leading to incomplete fermentation and a reduction in the volume of steamed bread. These issues arise because dietary fibers can alter the secondary structure or aggregation of gluten, thereby affecting dough quality and the final product. Specifically, dietary fiber can disrupt the gluten network by altering the intermolecular interactions between gluten proteins, which in turn changes the rheological properties of the dough and impacts product quality [3]. However, modifying dietary fibers can improve dough processing characteristics. Qingyang et al. [4] demonstrated that modifying dietary fibers with enzymes such as xylanase, cellulase, and amylase helped promote gluten protein polymerization and strengthened the gluten network, leading to high-fiber bakery products with better texture and palatability. Šoronja-Simović et al. [5] modified beet dietary fiber with hydrogen peroxide (H_2_O_2_), which not only significantly improved the brightness of the fiber, but also effectively changed its hydration properties. Wang et al. [6] employed steam blasting, a physical modification method, to enhance the rheological properties of rice bran dietary fibers, resulting in naan with improved specific volume, texture, and flavor. Similarly, Meththa et al. [7] used ultrasonic technology to modify date seed dietary fiber, improving the nutritional value of biscuits without negatively affecting consumer acceptance. The modified biscuits also showed delayed lipid oxidation during storage, better retention of phenolic compounds, and enhanced bioaccessibility of polyphenols after simulated digestion. Despite these advancements, there has been limited research on the mechanisms by which modified soybean residue dietary fiber affects dough processing characteristics. Further studies are needed to explore how modifications to soybean residue dietary fiber influence dough quality and the production of steamed bread. This would provide a deeper understanding of the underlying mechanisms and aid in the development of high-fiber, high-quality bakery products.

We hypothesize that modified soybean residue dietary fiber may influence dough processing characteristics and steamed bread quality by affecting gluten structure and its ability to bind water molecules. In this study, high-speed homogenization combined with enzymes (HEDF) and UEDF were used to prepare dietary fiber from soybean residues. These modified fibers were then incorporated into dough by replacing wheat flour at different ratios (4%, 6%, and 8%). The impact of these modified dietary fibers on dough and steamed bread quality was investigated through an analysis of the dough’s textural and rheological properties, the secondary structure of gluten proteins, the content of free hydrophobic groups and disulfide bonds, moisture distribution, and the microstructure of gluten proteins. This study provides a theoretical foundation for using dietary fiber to improve both the processing characteristics and nutritional quality of dough and steamed bread.

## 2. Materials and Methods

### 2.1. Materials

Wheat flour (16% protein, 74% carbohydrates, and 3% fat) was sourced from Wuteli Flour Group Co., Ltd. (Henan Province, China). Active dry yeast was purchased from Angie’s Yeast Co., Ltd. (Yichang, Hubei, China). Soybean residue was obtained from Yuxiang Bean Products Co., Ltd. in Changchun City, Jilin Province, China. All chemicals used were of analytical grade.

### 2.2. Modification and Extraction of Dietary Fiber from Soybean Residues

#### 2.2.1. Extraction Methods

Referring to Tian et al. [8] with slight modifications, the pH of the soybean residue sample suspension was adjusted to 6.0 and α-amylase (100 U/g) was added. The mixture was stirred at 95 °C for 30 min. The pH was then adjusted to 7.0 and papain (100 U/g) was added, with the suspension reacting at 55 °C for 30 min. Next, the pH was adjusted to 4.2 and the suspension was treated with amyloglucosidase (200 U/g) at 60 °C for 30 min. After completing the enzyme treatments, the enzymes were inactivated by boiling the mixture in a water bath for 5 min and then cooling it to room temperature. The enzyme solution was centrifuged at 6000 r/min for 15 min, and the precipitate was collected and freeze-dried to obtain insoluble dietary fiber. The supernatant was mixed with four times its volume of 95% ethanol and allowed to settle for 12 h. After settling, the mixture was centrifuged at 4000 r/min for 10 min. The filtrate was collected, freeze-dried, ground, and passed through a 100-mesh sieve. The insoluble dietary fiber and soluble dietary fiber obtained above were combined to obtain the total dietary fiber. The resulting samples were named unmodified dietary fiber (ODF), HEDF, and UEDF based on the respective modification processes.

#### 2.2.2. Modification Methods

Slightly modified from the method of Lin et al. [9], five grams of soybean residue dietary fiber was weighed and mixed with distilled water at a liquid-to-material ratio of 30:1 (mL/g). The mixture was then homogenized for 15 min at a high-speed homogenization rate of 10,000 r/min. Meanwhile, five grams of soybean residue dietary fiber was weighed and mixed with distilled water at a liquid-to-material ratio of 30:1 (mL/g). The mixture was then treated with ultrasound at 500 W power for 30 min. The dietary fiber samples from both modification methods were adjusted to pH 5.0 using 0.1 mol/L HCl. A composite enzyme solution (cellulase: xylanase ratio of 1:1) was added at 6% of the total mass and the mixture was enzymatically digested at 50 °C for 2 h. After the enzymatic digestion, the enzyme activity was terminated by boiling the samples in a water bath for 5 min.

### 2.3. Wheat Dough Preparation

The dough preparation referred to Liu et al. [10], with slight modifications. The dough recipe included 200 g of wheat flour and dietary fiber (DF). The moisture content was adjusted based on the fiber content obtained from the Mixolab testing results (Mixolab data included in the Supplementary Materials). The amount of dietary fiber added was based on the weight of the wheat flour and varied across four groups: 0%, 4%, 6%, and 8%. The preparation steps were as follows: first, activate the dry yeast with warm water. Then, add the DF and yeast to the wheat flour and mix with the required amount of distilled water. In a mixer, combine the ingredients at low speed for 2 min, then increase to high speed for 4 min until a smooth, homogeneous dough forms. The samples were labeled based on the type of modification and the percentage of DF added, as follows: unadded dietary fiber (WF dough), unmodified dietary fiber (ODF-4%, ODF-6%, and ODF-8%), high-speed homogenization combined with enzyme method (HEDF-4%, HEDF-6%, and HEDF-8%), and ultrasound combined with enzyme method (UEDF-4%, UEDF-6%, and UEDF-8%). Among them, the ODF group served as the control group.

### 2.4. Textural Properties of Wheat Dough

We used a texture analyzer to measure the textural characteristics of dough samples using Duan et al.’s [11] method (Load cell 5 kg, Honeywell Engineering, Morris Plains, NJ, USA). The prepared dough was cut into standard shapes of 0.2 cm × 1 cm × 3 cm for the TPA test. The textural properties (hardness, springiness, cohesiveness, and chewiness) of the dough samples were tested using a P/0.5R probe in TPA mode with the following parameter settings: pre-test speed of 1.0 mm/s; test speed of 1.0 mm/s; post-test speed of 1.0 mm/s; and a trigger force of 5.0 g with a strain of 60%. Six replications were performed for each measurement.

### 2.5. Measurement of Rheological Properties

#### 2.5.1. Rheological Performance Test

Frequency sweep measurements were investigated following the method of Meththa et al. [12] with minor modifications. Amplitude scanning was performed to determine the linear viscoelastic region. At a frequency of 1 Hz, the shear stress is within the range of 0.01–100% (the data are included in the Supplementary Materials). The test conditions were as follows: constant strain—0.04%; gap—2 mm; temperature—25 °C; and frequency sweep range—0.1–10 Hz. The storage modulus (G′), loss modulus (G″), and tanδ of the doughs were recorded (G″/G′).

#### 2.5.2. Creep Recovery Measurement

Creep properties were measured following Shan et al. [13]. Frequency sweep measurements were conducted following the method of Meththa et al. [12]. The test conditions were as follows: a constant stress of 50 Pa applied for 60 s, followed by a recovery period of 120 s after stress relief. The clearance was set to 2 mm and the test was conducted at a temperature of 25 °C. Creep strain (maximum creep strain) and recovery strain were recorded.

### 2.6. Fourier Transform Infrared Spectroscopy (FT-IR) Analysis

The secondary structure content of the samples was determined using FT-IR (Spectrum 100, PerkinElmer Co., Ltd., Waltham, MA, USA) in reference to a slight modification of the method of Yu et al. [14]. The freeze-dried dough samples were ground in an agate mortar with KBr powder at a ratio of 1:100 and then pressed into tablets prior to analysis. The range of the scanned spectra was set from 600 to 4000 cm^−1^, with a resolution of 4 cm^−1^ and a scanning frequency of 64 scans. Second-order derivative analysis and curve fitting were performed using OMNIC and Peak Fit 4.12 software to assess the secondary structure of the samples.

### 2.7. Determination of Free Sulfhydryl and Disulfide Bond

#### 2.7.1. Determination of Free Sulfhydryl

Determination was performed using the method of Yu et al. [14]. First, 0.05 g of dough samples was accurately weighed into a centrifuge tube and 20 mL of 0.2 mol/L Tris-Gly buffer solution was added and gently stirred to dissolve it fully. Then, the proteins were centrifuged at 2000 r/min for 5 min to separate the proteins moderately and to avoid protein denaturation due to excessive manipulation. Next, 2 mL of supernatant was taken, and 0.2 mL of 10 mmol/L (5,5′-dithiobis-2-nitrobenzoic acid) DTNB solution was added, shaken gently, and left at room temperature for 30 min. The absorbance was then measured at 412 nm. The free sulfhydryl content was calculated by substituting the measured absorbance into a pre-drawn standard curve using the formula

$$\mu \mathrm{mol}\;\mathrm{SH}/g=\frac{{73.53A}_{412}\times D}{C}$$

where, in the formula 73.53 = 106/(1.36 × 104), 1.36 × 104 is the molar extinction coefficient of the DTNB solution; A_412_ is the absorbance value of the sample at a wavelength of 412 nm; D is the dilution factor, where D = 5; C is the sample mass concentration in mg solid/mL units.

#### 2.7.2. Determination of the Disulfide Bond

0.5 mL of the above supernatant was accurately aspirated, 0.1 mL of mercaptoethanol and 3 mL of buffer were added, and the mixture was held at 25 °C for 1 h. Next, the supernatant was removed by centrifugation at 4000 r/min for 15 min, and the precipitate was washed twice with 5 mL of 12% trichloroacetic acid to remove impurities adequately. Tris-glycine-EDTA buffer (TGE) consisted of 92 mmol/L glycine, 4.1 mmol/L EDTA, and 86 mmol/L Tris-HCl (pH 8.0). Subsequently, the precipitate was dissolved in 8 mL of buffer and 2 mL of the dissolved solution was aspirated. Then, 2 mL of buffer and 0.1 mL of 10 mmol/L DTNB solution were added and the mixture was mixed well and allowed to develop color in the dark for 20 min. Finally, the absorbance was measured at 412 nm. The content of disulfide bonds was calculated by substituting the measured absorbance into the formula derived from previous experimental data.

Total mercapto content (μmol/g) = Y × N/M

Disulfide bond content (μmol/g) = (total mercapto content − free mercapto content)/2

where Y is total SH concentration in μmol/mL; N is the dilution factor (320); and M is the sample mass (g).

### 2.8. Low-Field Nuclear Magnetic Resonance (LF-NMR)

The moisture distribution of the dough was measured using a low-field NMR analyzer (VTMR20-010 V-1, Newmark Analytical Instruments Co., Ltd., Shanghai, China), according to the method reported by Lu et al. [15], with slight modifications. The dough sample (1.5 g) was placed in an NMR cuvette and then put in the instrument sampling position. Finally, the analysis was carried out under the following conditions: SW = 200 kHz, TW = 300 ms, NECH = 2000, TE = 0.18 ms, and NS = 64. The minimum relaxation time was 0.01 ms, the maximum relaxation time was 10,000 ms, and the value of iterations was 100,000. Three replications were performed for each determination.

### 2.9. Scanning Electron Microscope (SEM)

Dough samples were analyzed for microstructure using a scanning electron microscope (SU1510, Hitachi High-Tech Corporation, Tokyo, Japan) according to the method of Almoumen et al. [16]. The freeze-dried samples were fixed on a unique platform using conductive adhesive for 40 s of gold spraying. The working voltage was set to 20 kV and the magnification to 500× and 1000× to obtain high-resolution microstructural images of the dough samples.

### 2.10. Steamed Bread Preparation

The dough preparation process was the same as in 2.2, in which 6% soybean residue dietary fiber addition was selected. After dough preparation, it was placed in a rising box with 80% relative humidity and steamed for 20 min after fermentation for 1 h. A resulting composite steamed bread with different modified DFs was obtained. Before all analyses, the steamed bread was cooled at room temperature for 1 h. The steamed bread was named WS, WS-ODF, WS-HEDF, and WS-UEDF according to the different modifications.

### 2.11. Measurement of Specific Volume and Height-to-Diameter Ratio of Steamed Bread

Specific volume determination method: Determine the quality of cooled steamed bread, use the millet substitution method to determine the volume of steamed bread, and repeat 3 times to take the average value [17]. The specific volume is calculated using the following formula:

(1) $$\mathrm{specific}\;\mathrm{volume}\;(\mathrm{mL}/g)=\frac{\mathrm{volumetric}\;(\mathrm{mL})}{\mathrm{weight}\;(g)}$$

Measurement method of height–diameter ratio: Measure the diameter and height of the steamed bread with electronic vernier calipers, calculate the height–diameter ratio, and repeat 3 times to take the average value. The following formula is used to calculate the height–diameter ratio:

(2) $$\mathrm{aspect}\;\mathrm{ratio}=\frac{\mathrm{height}\;(\mathrm{mm})}{\mathrm{diameter}\;(\mathrm{mm})}$$

### 2.12. Measurement of the Texture of Steamed Bread

The texture characteristics of steamed bread samples were measured using a texture analyzer (weighing sensor 5 kg, Load cell 5 kg, Honeywell Engineering, Morris Plains, NJ, USA). The probe type was the same as the dough. We cut the steamed bread into pieces 2 cm wide, 4 cm long, and 5 cm high for measurement. The pre-measurement speed was 1.0 mm/s, the post-measurement speed was 1.0 mm/s, and the trigger force was 5.0 g. The deformation level was 50% of the sample height. The metrics determined by this test included hardness, springiness, cohesiveness, chewiness, viscosity, and resilience. The test was completed in three repetitions.

### 2.13. Color Analysis of Steamed Bread Crumb

This was modified according to Ma et al.’s [18] method. We conduct three measurements on each steamed bread crumb sample and expressed them in the CIE-L*a*b* color space, where L* indicates brightness (on a lightness–darkness scale). Chromaticity was expressed as follows: L* coordinates from 0 (dark) to 100 (light); a* coordinates from 60 (red) to −60 (green); and b* coordinates from 60 (yellow) to −60 (blue). The colorimeter was calibrated with a white standard (L* = 97.21; b* = 0.14; and a* = 1.99). Notably, the total color difference (ΔE) between the samples was relative to the color difference between the control sample and the test samples.

$$∆E=\sqrt{{∆L}^{*2}+{∆a}^{*2}{+∆b}^{*2}}$$

### 2.14. Sensory Evaluation of Steamed Bread

This was based on the evaluation method of Beibei et al. [19], with appropriate adjustments. Ten sensory assessors with professional backgrounds were selected to conduct sensory assessments of steamed bread quality. The evaluation indexes covered external integrity, surface smoothness, specific volume, internal uniformity, chewiness, elasticity, viscosity, and the odor of the steamed bread, among others. The specific evaluation criteria are shown in Table 1. Each evaluator scored independently, and the final evaluation result used the average value of all the scores to ensure the objectivity and accuracy of the evaluation.

### 2.15. Statistical Analysis

All measurements were replicated three times. IBM SPSS 26 (International Business Machines Corporation, Armonk, NY, USA) and Origin 2022 (Origin Lab Corporation, Northampton, MA, USA) were used for data analysis and graphing. The data were presented as the mean value ± standard deviation. Differences between the experimental groups were significant at the level of *p* < 0.05 in this work.

## 3. Results and Discussion

### 3.1. Texture Property Test

The data in Table 2 show that the addition and modification of soybean dietary fiber significantly influenced the textural properties (TPA parameters) of the dough. As the dietary fiber content increased from 4% to 8%, both the hardness and chewiness of the dough gradually increased, while the elasticity and cohesiveness initially increased and then decreased. This pattern can be attributed to the competition between dietary fiber and gluten proteins for water, which hinders the formation of the gluten network. As DF absorbs water and swells, it crosslinks with the gluten proteins, resulting in a tougher, finer spatial structure that improves the dough’s elasticity and cohesiveness. This finding aligns with the results of Jiang et al. [20]. However, excessive DF addition may reduce the water absorption capacity of gluten proteins, leading to localized dehydration, which can impair the dough’s elasticity and cohesiveness, as well as decrease protein content and hinder water uptake by gluten proteins [21].

The dough in the HEDF-6% and UEDF-6% groups showed a significant reduction in hardness and chewiness, with decreases of 263.24N and 267.17N for HEDF-6% and 179.11N and 126.68N for UEDF-6%. Conversely, the elasticity of these doughs increased, rising from 0.81 in the ODF-6% group to 0.82 in the HEDF-6% group and 0.89 in the UEDF-6% group. This improvement in elasticity can be attributed to the modification methods used. These modifications enhanced the water-holding capacity of the fibers, making the dough more malleable and improving its elasticity. The enhanced water-binding properties of the dietary fibers, starch, and gluten likely contributed to this effect [22].

Overall, the incorporation of HEDF and UEDF improved the dough’s viscosity, elasticity, water absorption, stability, and texture. Additionally, these modifications increased the nutritional value of the dough. However, it is important to control the amount of dietary fiber added to prevent negative effects on dough texture and mouthfeel. These findings provide a deeper understanding of how soybean dietary fiber and its modifications impact dough texture properties.

### 3.2. Rheological Properties

#### 3.2.1. Rheological Characterization

**Figure 1:**
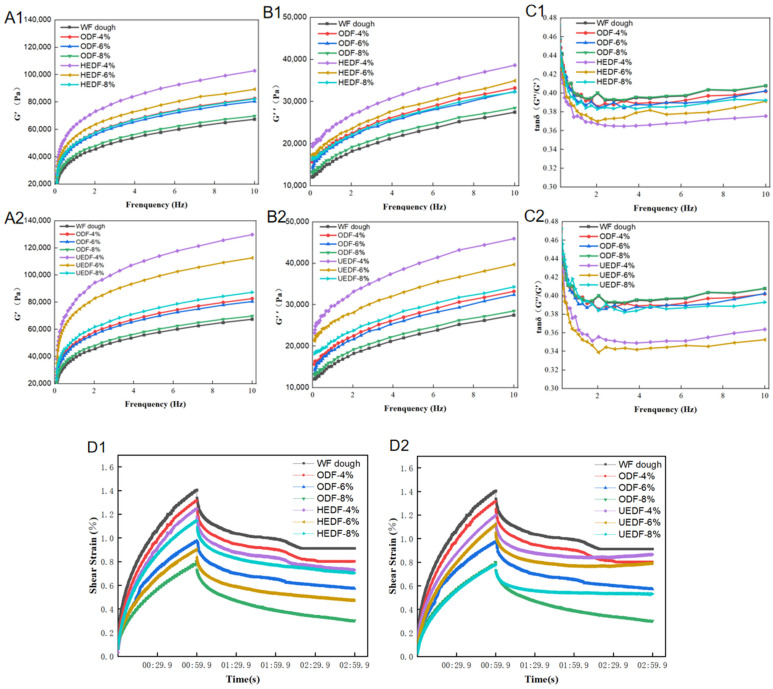
Dynamic rheology (**A**–**C**) and creep recovery measurements (**D**) of composite dough with different modified dietary fibers. (**A**) Variation of energy storage modulus (G′) at various frequencies. (**B**) Variation of loss modulus (G″) at different frequencies. (**C**) Variation of G′/G″ (G″) ratio at various frequencies. (**D**) Creep recovery measurement.

Rheological characterization of dough is essential for analyzing its viscoelastic properties during processing and baking. As shown in Figure 1A–C, both the energy storage modulus (G′) and the loss modulus (G″) increased with frequency in the range of 0.1–10 Hz, with the G′ value consistently higher than G″. This indicates that the dough exhibited stronger elastic interactions, suggesting a more robust dough structure. The tanδ (G″/G′) values for all the dough samples remained below 1, indicating that the dough primarily exhibited solid-like elastic behavior [23].

For most dough samples, the addition of modified dietary fiber resulted in a significant increase in both G′ and G″. These findings align with previous research by Li et al. [24]. Notably, the dough samples from the UEDF group showed the most pronounced changes. This suggests that the modified dietary fibers more effectively enhance interactions with starch molecules in the dough, forming a tighter and stronger network [25]. However, it is important to note that when dietary fiber is added in excess, both the storage modulus (G′) and loss modulus (G″) decrease significantly. This may occur because excessive dietary fiber can disrupt disulfide bonds, weakening some of the gluten crosslinking, which in turn reduces the dough’s flow and elasticity [26].

#### 3.2.2. Creep Recovery Measurement Results

As shown in Figure 1D, the creep recovery curve of the dough consists of two phases: creep and recovery. During the creep phase, a constant force is applied to the dough, allowing it to reach a steady state over a specified period. The recovery phase characterizes the dough’s viscoelastic behavior over a longer duration [27].

The effect of the UEDF group on the creep strain of the dough followed a consistent pattern: creep strain decreased as the dietary fiber content increased. Chong-Chong et al. [28] noted that creep strain is related to the moisture content of the dough, with doughs containing higher moisture exhibiting more significant maximum creep strain. The reduction in creep strain in this study suggests improved deformation resistance in the dietary fiber-enriched doughs. This could be due to the ability of modified dietary fibers to promote hydrogen bond formation within the dough, enhancing its gel-forming properties. This finding is in line with the results of Dangi et al. [29], who added natural and partially hydrolyzed β-glucan concentrates to rice flour doughs, improving their resistance to deformation.

### 3.3. FT-IR Analysis

**Figure 2:**
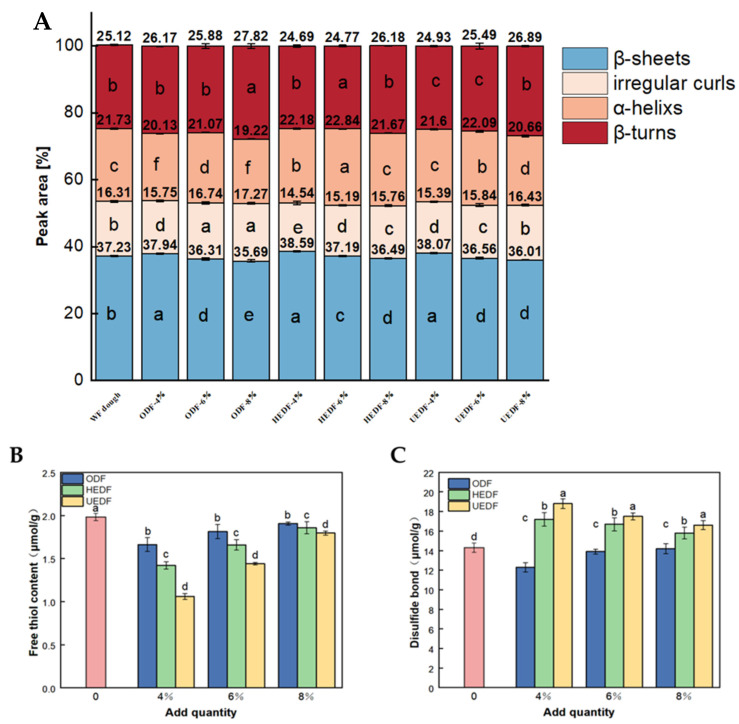
Changes in gluten protein secondary structure (**A**), free sulfhydryl groups (**B**), and disulfide bond content (**C**) by different modified dietary fibers (different lowercase letters on the same legend in the figure indicate significant differences at the *p* < 0.05 level).

Fourier transform infrared spectroscopy is commonly used to analyze changes in the secondary structures of gluten proteins in dough. The amide I band (1600–1700 cm^−1^) is particularly sensitive to such changes and is frequently used to assess protein structure. Within this range, 1600–1640 cm^−1^ corresponds to β-folding, 1640–1650 cm^−1^ to irregular curling, 1650–1660 cm^−1^ to α-helix, and 1660–1700 cm^−1^ to β-turning. The β-fold and α-helix structures are among the most stable and ordered, suggesting a more stable protein structure, while irregular curls and β-turns indicate less ordered configurations [30]. As shown in Figure 2A, β-folding and β-turning were the primary forms of the secondary structure of gluten, consistent with the findings of Chen et al. [31].

Compared to the ODF-6% group, the β-fold content increased from 36.31% to 37.19% in the HEDF-6% group and 38.86% in the UEDF-6% group. This increase can be attributed to interactions, such as hydrogen bonding and hydrophobic interactions, between dietary fibers and gluten proteins, which promote the formation of β-folded structures. Feng et al. [32] also showed that hydrogen bonding stabilizes α-helix and β-folded structures, enhancing the orderly arrangement and tight packing of protein molecules. However, when the dietary fiber content increased from 6% to 8%, the β-fold content in ODF, HEDF, and UEDF decreased by 0.62%, 0.7%, and 2.85%, respectively, while irregular curls and β-turns increased significantly. This suggests that excessive dietary fiber disrupts hydrogen bonds in the β-folded structures, leading to a shift from ordered β-folding to less stable forms like irregular curls and α-helices. This phenomenon of gluten protein unfolding aligns with the findings of Bao et al. [33].

Overall, the addition of 6% modified soybean residue dietary fiber enhances the stability of the gluten protein network and promotes a more ordered protein structure. This improvement significantly enhances the dough’s processing characteristics, making it more stable and easier to handle during production.

### 3.4. Free Hydrophobic and Disulfide Bond Content

Free sulfhydryl groups and disulfide bonds are crucial functional groups in gluten proteins, influencing the degree of covalent bonding and crosslinking within the gluten network. Changes in the content of free sulfhydryl groups reflect alterations in the structure and stability of the gluten proteins, while disulfide bonds play a key role in maintaining their stability [34]. Together, these factors determine the formation of the gluten network, which directly impacts the final quality of the dough product.

As shown in Figure 2B, at a 4% addition of soybean residue dietary fiber, the highest content of free sulfhydryl groups was 1.66 μmol/g in the ODF group, while the lowest was 1.06 μmol/g in the UEDF group. At a 6% addition, the highest free sulfhydryl group content was 1.81 μmol/g in the ODF group, and the lowest was 1.44 μmol/g in the UEDF group. For the 8% addition, the highest was 1.91 μmol/g in the ODF group, and the lowest was 1.80 μmol/g in the UEDF group. Conversely, the content of disulfide bonds increased with dietary fiber addition, with the highest value being 18.80 μmol/g in the 4% UEDF group and the lowest 12.30 μmol/g in the 4% ODF group. This increase in disulfide bonds can be attributed to the strong water-holding capacity of the soybean residue dietary fibers, which enhances the compactness of the gluten protein network.

Similar findings were reported by Guo et al. [35], who observed that the addition of dietary fiber promoted hydrogen bonding interactions within gluten proteins, leading to a conversion of free sulfhydryl groups into disulfide bonds and accelerating protein aggregation. In this study, the enhanced crosslinking of disulfide bonds due to the modified soybean residue dietary fibers contributed to the formation of a denser gluten network. This structural adjustment is crucial for optimizing the quality of dough products, offering valuable insights into improving the processing characteristics of gluten-based products.

### 3.5. LF-NMR Analysis

**Figure 3:**
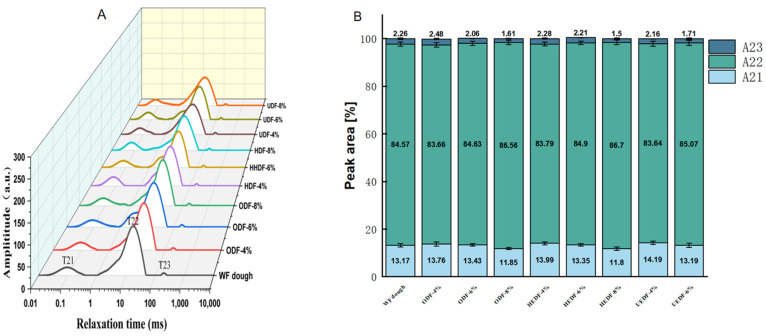
Moisture migration (**A**) and peak values (**B**) of different modified dietary fiber composite dough.

Moisture distribution in dough plays a crucial role in its processing characteristics. Based on the associated relaxation times, water in dough can be classified into three categories: T_21_ (0.1 ms to 1 ms) for strongly bound water, T_22_ (1 ms to 50 ms) for weakly bound water, and T_23_ (50 ms to 300 ms) for free water [27]. As shown in Table 3 and Figure 3, the addition of modified dietary fiber caused the T_21_ relaxation time to shift leftward, with the UEDF group showing the most significant change. The T_21_ relaxation time decreased from 0.12 ms to 0.06 ms, which can be attributed to the enhanced water absorption capacity and increased affinity of the modified dietary fiber for water molecules. This interaction reduces the mobility of water molecules, resulting in a shorter relaxation time. This finding is consistent with Xie et al. [36], who observed that a higher SDF/IDF ratio in soybean residue decreased the fluidity of water in noodles.

The relaxation peak area also showed significant changes, particularly in the UEDF group. The area for free water (A_23_) decreased from 2.48% to 1.28%, while the area for weakly bound water (A_22_) increased from 83.66% to 86.65%. These changes suggest that the introduction of modified dietary fiber enhanced the dough’s water absorption capacity, leading to more water molecules being absorbed by the fiber and transitioning from free water to bound water. Chong-Chong et al. [28] similarly found that adding insoluble dietary fiber and ferulic acid resulted in a more even water distribution in dough, reducing the space available for free water. This combination of factors led to a decrease in free water and an increase in bound water.

The increased bound water content may be due to the hydrophilic groups in the modified dietary fibers, which compete with gluten and starch particles for water during dough formation. Additionally, the spatial arrangement of these fibers may disrupt hydrogen-bonding interactions between gluten proteins, starch molecules, and water, hindering the formation of a gluten network. This disruption reduces free water content and increases the amount of bound water in dough [37].

### 3.6. SEM Analysis

**Figure 4:**
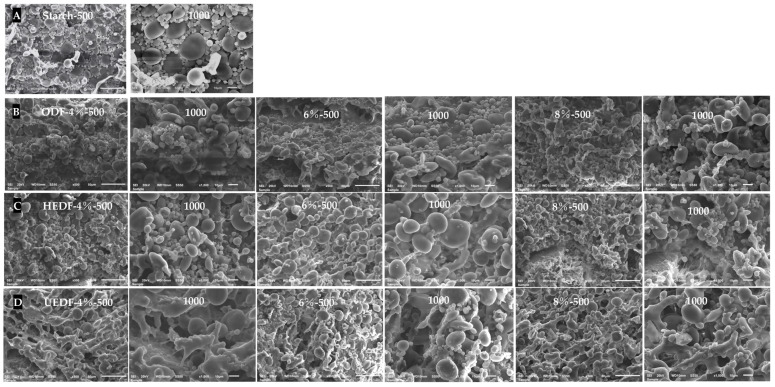
SEM images of wheat dough showing the effects of modified dietary fiber: ((**A**) starch, (**B**) ODF, (**C**) HEDF, and (**D**) UEDF).

Scanning electron microscopy was used to observe the microstructure of dough gluten proteins, revealing the typical three-dimensional network structure of gluten embedded with starch granules of various sizes and shapes [38]. In doughs without soybean residue dietary fiber, the gluten network exhibited unevenly sized interstices, with many starch granules exposed outside the gluten matrix (Figure 4A). The addition of unmodified soybean residue dietary fiber resulted in some structural disruption, although the fracture morphology was not clearly defined (Figure 4B). In the HEDF group, a significant number of starch granules remained exposed, but the continuity of the gluten network was improved, suggesting that HEDF enhanced the dough’s structural integrity (Figure 4C).

In contrast, the UEDF group displayed a more continuous and compact gluten network with fewer exposed starch granules and more uniform pore distribution (Figure 4D). This suggests that moderate supplementation with UEDF allowed more starch granules to be embedded in the gluten network, helping to protect the gluten structure from mechanical damage. It is hypothesized that interactions between modified dietary fibers and starch form complexes that fill pores or alter protein structures [39]. Zhang et al. [40] found that soluble dietary fibers from rice bran could adsorb water molecules via hydrogen bonding, inhibiting ice crystal growth and enhancing the stability of the gluten structure and the dough’s ability to hold air. These microstructural changes may significantly impact dough’s processing properties and final product quality. For instance, they could influence dough extensibility, softness, and the texture and specific volume of steamed bread.

### 3.7. Height and Specific Volume of Steamed Bread

**Figure 5:**
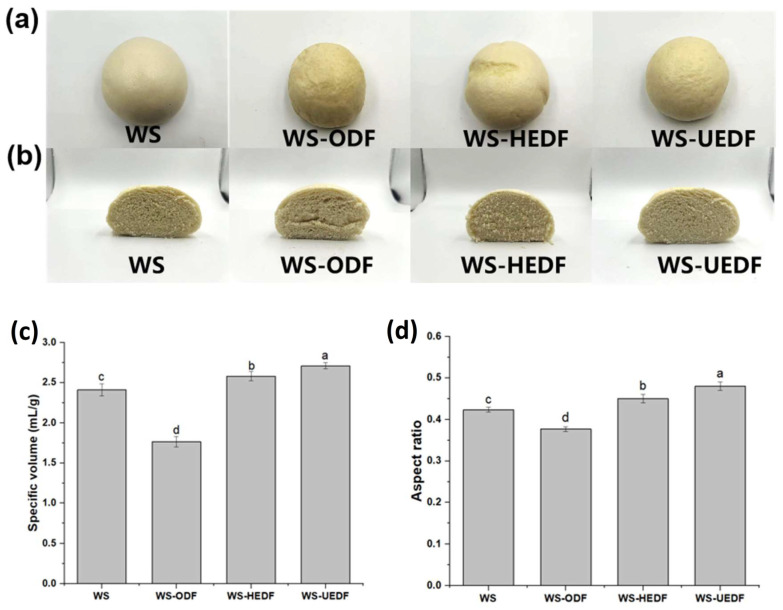
(**a**) Sliced cross-section, (**b**) top view, (**c**) specific volume, and (**d**) height-to-diameter ratio of composite steamed bread with different modified dietary fibers (different lowercase letters on the same legend in the figure indicate significant differences at the *p* < 0.05 level).

Figure 5a,b shows the top view and sliced cross-section of steamed bread made with different modified soybean residue dietary fibers. Specific volume is a key factor in evaluating the fluffiness of steamed bread during the baking process [41]. The specific volumes and height-to-diameter ratios of steamed bread with various modified DFs are presented in Figure 5c,d. The specific volume of steamed bread without added DF was 2.41 mL/g. When unmodified DF was added, the specific volume decreased from 2.41 mL/g to 1.76 mL/g compared to the control, indicating that unmodified DF reduced the specific volume and possibly caused the collapse of the steamed bread. This is likely due to the unmodified DF disrupting disulfide bonds in the gluten proteins, which weakens the gluten network and leads to a denser structure, thereby reducing the bread’s volume. These findings are consistent with those of Krekora et al. [42], who observed that phenolic acids could break disulfide bonds in gluten proteins, preventing the dough from rising and expanding properly.

In contrast, the addition of HEDF and UEDF improved the specific volume of the steamed bread, increasing it from 2.41 mL/g to 2.58 mL/g and 2.71 mL/g, respectively. This suggests that UEDF enhanced the dough’s gas-holding capacity during fermentation, allowing CO_2_ to be better retained within the dough. Zhao et al. [43] similarly found that the addition of konjac glucomannan helped form a stable starch–gluten matrix, ensuring uniform expansion of air pockets. Furthermore, the height-to-diameter ratio, which reflects the steamed bread’s morphology, followed similar trends to the specific volume changes, as shown in Figure 5d.

### 3.8. Textural Properties of Steamed Bread

Texture analysis is an essential method for evaluating food quality, particularly in steamed bread where texture significantly influences flavor—a key factor for consumer preference [44]. Table 4 presents the texture analysis results for soybean residue dietary fiber composite steamed bread slices after cooling for 1 h. The analysis covered six indicators: hardness, elasticity, stickiness, chewiness, cohesiveness, and reparability. Among these, elasticity, cohesiveness, and reparability are positively correlated with steamed bread quality, while hardness, chewiness, and stickiness are negatively correlated.

As shown in Table 4, the addition of DF resulted in increased hardness, chewiness, and stickiness, while elasticity, cohesiveness, and recovery were reduced. This effect is likely due to the strong hydrogen bonding interactions between DF and gluten proteins, which disrupt the continuity and strength of the gluten network. Consequently, the dough becomes harder, and its elasticity decreases [45]. The degree of expansion and internal stability of steamed bread is reflected in its elasticity, cohesiveness, and recovery. Compared to the ODF group, the hardness and chewiness of steamed bread in the HEDF and UEDF groups decreased by 359.18N and 183.56N, and 444.53N and 236.94N, respectively. In contrast, elasticity increased from 0.70 to 0.77 in the HEDF group and 0.82 in the UEDF group. This improvement can be attributed to the modified soybean residues’ dietary fiber, which enhanced the dough’s water absorption capacity. The increased water content helped loosen the gluten network, making the steamed bread less firm. Similar results were reported by Bai et al. [46], who found that adding 4% red kidney bean polysaccharides strengthened the gluten network and improved the elasticity of steamed bread, likely due to the enhanced viscosity and elasticity of the dough.

### 3.9. Color of Steamed Bread

Color is a key factor in consumer perception and serves as an important physical indicator of food quality, particularly in steamed bread [47]. Table 5 presents the effects of different levels of modified soybean residue dietary fibers on the color characteristics of steamed bread. Compared to the whole steamed bread group, the L* value in the ODF group decreased by 13.65, while the a* and b* values increased by 4.98 and 10.73, respectively. This change suggests that the darker components in soybean residues contributed to a deeper color in the steamed bread. Similar findings were reported in previous studies; for instance, Luo et al. [48] observed that inulin and dough undergo a Maillard reaction during steaming, which can darken the product. However, since steaming temperatures are typically lower than baking temperatures, the extent of non-enzymatic browning is reduced, leading to a less intense color change.

In the HEDF and UEDF groups, the L* value increased by 2.97 and 4.69, respectively, compared to the ODF group. This suggests that the modification of dietary fibers enhanced their structure, improving their distribution and homogeneity within the dough. This structural improvement may contribute to better light reflection and, consequently, a brighter appearance in the steamed bread [49].

### 3.10. Sensory Evaluation

**Figure 6:**
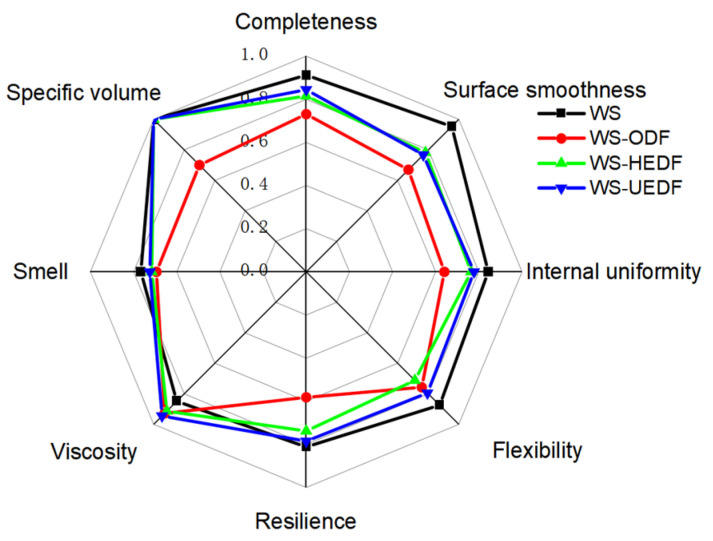
Sensory evaluation radar chart.

As shown in Figure 6 and Table 6, the overall sensory scores for steamed bread containing unmodified soybean residue dietary fiber were significantly lower compared to steamed bread without added DF. This was primarily due to the coarse texture and strong soy flavor of the unmodified soybean residue fiber, which contributed to a dry, less chewy texture and an unpleasant soy odor. Additionally, the unmodified fiber had a darker color and was difficult to disperse evenly, leading to inconsistencies in the steamed bread’s appearance and visual appeal.

In contrast, the composite steamed bread made with modified dietary fiber met national standards for specific volume and exhibited improved texture, with better scores for gluten, elasticity, toughness, and viscosity. The steamed bread without added DF and with no added fiber received the highest sensory score of 86.26, while the steamed bread with unmodified dietary fiber scored the lowest at 62.51. Both the WS-HEDF and WS-UEDF groups scored over 80, with the WS-UEDF group most closely resembling the steamed bread without added DF in terms of sensory attributes.

These results demonstrate that combining ultrasound and enzyme modification techniques effectively improves the texture, reduces the undesirable odor, enhances color consistency, and optimizes the expansion and pore structure of the steamed bread. The UEDF group, in particular, produced steamed bread with sensory qualities similar to steamed bread without added DF, including taste, aroma, and visual appeal. This highlights the significant benefits of ultrasound and enzyme modifications in enhancing the utilization of soybean residue dietary fiber for pasta production.

## 4. Conclusions

In this study, HEDF and UEDF, as substitutes for wheat flour, significantly improved the processing characteristics of dough and the quality of soybean residue dietary fiber steamed bread. Specifically, using untreated fiber samples as the control group, these two modified dietary fibers increase the proportion of beta folding structures and disulfide bond content in gluten, thereby improving the water retention capacity and structural stability of the dough. In particular, the dough containing 6% soybean residue dietary fiber exhibits the best processing characteristics. The steamed bread prepared with 6% UEDF has good viscoelasticity, specific volume, and sensory evaluation. However, there are potential limitations to the effect of adding modified soybean residue dietary fiber on dough extensibility and fermentation performance. Future research should further optimize the fiber modification process and explore its application prospects in other food systems.

## Figures and Tables

**Table 1:** Steamed bread evaluation criteria table.

Attribute	Marking Scheme	Value of a Score
Completeness	Complete shape, no cracks on the surface	6–10
	Incomplete shape with surface cracks	0–5
Surface smoothness	The surface is very smooth, with no wrinkles, no bubbles or hard lumps	7–10
	Smooth surface, a few folds, no bubbles or hard lumps	4–6
	The surface is not smooth, wrinkles are obvious, there are bubbles and hard lumps	0–3
Internal uniformity	The stomata are fine and evenly distributed, with good layering and no large holes	12–15
	Stomata are fine and more evenly distributed, better layered, and have slightly larger pores	8–11
	Uneven porosity, rough structure, more large pores	0–7
Flexibility	Bite-sized, chewy, no crumbs	7–10
	More substantial, more chewy, not easy to fall out of the crumbs	4–6
	Poor bite, very easy to fall out	0–3
Resilience	Soft, elastic, immediate recovery after pressing, fast rebound	16–20
	Soft, slow recovery after pressing, slow rebound	10–15
Viscosity	Chewable and non-sticky	7–10
	medium	4–6
	Unpleasant to chew, sticky teeth	0–3
Smell	Wheat aroma, bean aroma, no bad taste	12–15
	Bland flavor, slightly off flavor There is an odor	9–11 0–8
Specific volume	2.30 mL/g full marks, 1 mark for every 0.2 less	0–10

**Table 2:** Textural analysis of composite dough with different modified dietary fibers.

Samples	Hardness/N	Springiness	Chewiness/N	Cohesiveness
WF-dough	765.64 ± 13.28 ^e^	0.72 ± 0.02 ^e^	380.36 ± 9.45 ^h^	0.69 ± 0.03 ^bc^
ODF-4%	1265.64 ± 13.28 ^e^	0.87 ± 0.02 ^bc^	781.78 ± 9.45 ^f^	0.71 ± 0.03 ^bc^
ODF-6%	1738.76 ± 13.39 ^b^	0.81 ± 0.02 ^d^	1098.54 ± 23.78 ^b^	0.78 ± 0.02 ^a^
ODF-8%	1938.73 ± 17.54 ^a^	0.76 ± 0.01 ^e^	1134.54 ± 16.81 ^a^	0.77 ± 0.01 ^a^
HEDF-4%	1164.10 ± 12.49 ^f^	0.89 ± 0.03 ^ab^	621.62 ± 23.78 ^g^	0.60 ± 0.03 ^f^
HEDF-6%	1475.52 ± 10.15 ^d^	0.82 ± 0.03 ^cd^	919.43 ± 16.90 ^d^	0.76 ± 0.03 ^a^
HEDF-8%	1640.09 ± 16.65 ^c^	0.79 ± 0.02 ^d^	816.27 ± 18.47 ^e^	0.63 ± 0.02 ^f^
UEDF-4%	1245.85 ± 12.70 ^e^	0.92 ± 0.03 ^a^	631.88 ± 14.11 ^g^	0.70 ± 0.02 ^bc^
UEDF-6%	1471.59 ± 19.89 ^d^	0.89 ± 0.09 ^ab^	971.86 ± 223.03 ^c^	0.74 ± 0.03 ^ab^
UEDF-8%	1651.73 ± 15.60 ^c^	0.81 ± 0.02 ^d^	896.39 ± 16.59 ^d^	0.67 ± 0.01 ^cd^

Values in the same row with different superscripts are significantly different (*p* < 0.05).

**Table 3:** Moisture distribution of composite dough with different modified dietary fibers.

Samples	Relaxation Time [ms]			Peak Area [%]		
	T21	T22	T23	A21	A22	A23
WF dough	0.12 ± 0.003 ^a^	18.32 ± 0.21 ^a^	228.14 ± 3.41 ^a^	13.17 ± 0.16 ^b^	84.57 ± 2.03 ^c^	2.26 ± 0.03 ^a^
ODF-4%	0.10 ± 0.002 ^a^	18.11 ± 0.45 ^a^	224.31 ± 4.73 ^a^	13.76 ± 0.25 ^b^	83.66 ± 1.41 ^c^	2.48 ± 0.06 ^a^
ODF-6%	0.09 ± 0.01 ^b^	18.04 ± 0.55 ^a^	215.52 ± 4.51 ^b^	13.43 ± 0.05 ^c^	84.63 ± 2.15 ^b^	2.06 ± 0.05 ^b^
ODF-8%	0.08 ± 0.004 ^b^	16.61 ± 0.53 ^b^	202.30 ± 3.76 ^e^	11.85 ± 0.13 ^e^	86.56 ± 3.20 ^a^	1.61 ± 0.02 ^d^
HEDF-4%	0.08 ± 0.003 ^b^	15.60 ± 0.46 ^d^	217.34 ± 2.75 ^b^	13.99 ± 0.03 ^a^	83.79 ± 2.12 ^c^	2.28 ± 0.02 ^a^
HEDF-6%	0.07 ± 0.004 ^c^	16.67 ± 0.42 ^b^	210.39 ± 1.21 ^c^	13.35 ± 0.21 ^d^	84.90 ± 3.14 ^b^	2.21 ± 0.10 ^c^
HEDF-8%	0.07 ± 0.003 ^c^	13.61 ± 0.91 ^c^	197.50 ± 2.17 ^f^	11.80 ± 0.40 ^e^	86.70 ± 4.11 ^a^	1.52 ± 0.03 ^d^
UEDF-4%	0.08 ± 0.004 ^b^	15.46 ± 0.40 ^c^	214.43 ± 2.12 ^b^	14.19 ± 0.07 ^a^	83.64 ± 3.06 ^c^	2.16 ± 0.05 ^b^
UEDF-6%	0.07 ± 0.003 ^c^	16.55 ± 0.48 ^b^	207.94 ± 2.54 ^d^	13.19 ± 0.08 ^d^	85.07 ± 2.08 ^b^	1.71 ± 0.03 ^d^
UEDF-8%	0.06 ± 0.006 ^d^	16.88 ± 0.10 ^b^	193.10 ± 3.68 ^f^	12.07 ± 0.10 ^e^	86.65 ± 1.07 ^a^	1.28 ± 0.05 ^e^

Values in the same row with different superscripts are significantly different (*p* < 0.05).

**Table 4:** Properties of different modified dietary fiber composite steamed breads.

Samples	Hardness/N	Springiness	Chewiness/N	Viscosity/pa.s	Cohesiveness	Resilience
WS	566.66 ± 13.94 ^d^	0.88 ± 0.01 ^a^	440.65 ± 19.45 ^d^	498.74 ± 17.61 ^d^	0.82 ± 0.03 ^a^	0.49 ± 0.01 ^a^
WS-ODF	1508.47 ± 18.26 ^a^	0.70 ± 0.03 ^d^	964.60 ± 22.95 ^a^	735.57 ± 33.85 ^c^	0.64 ± 0.02 ^d^	0.28 ± 0.02 ^d^
WS-HEDF	1149.29 ± 18.68 ^c^	0.77 ± 0.04 ^c^	519.47 ± 12.78 ^c^	944.51 ± 8.77 ^a^	0.77 ± 0.05 ^b^	0.42 ± 0.05 ^b^
WS-UEDF	1324.91 ± 39.18 ^b^	0.82 ± 0.06 ^b^	727.66 ± 30.46 ^b^	1171.52 ± 26.94 ^b^	0.71 ± 0.07 ^c^	0.35 ± 0.05 ^c^

Values in the same row with different superscripts are significantly different (*p* < 0.05).

**Table 5:** Color of composite steamed bread with different modified dietary fibers.

Samples	L*	a*	b*	ΔE
WS	34.35 ± 0.25 ^a^	5.26 ± 0.12 ^d^	11.17 ± 0.21 ^d^	-
WS-ODF	20.70 ± 0.11 ^d^	10.22 ± 0.09 ^a^	21.90 ± 0.20 ^a^	18.05 ± 0.43 ^a^
WS-HEDF	23.36 ± 0.22 ^c^	6.49 ± 0.16 ^c^	16.52 ± 0.19 ^b^	12.28 ± 0.24 ^b^
WS-UEDF	25.39 ± 0.25 ^c^	8.32 ± 0.08 ^b^	15.08 ± 0.17 ^c^	10.24 ± 0.36 ^c^

Values in the same row with different superscripts are significantly different (*p* < 0.05).

**Table 6:** Sensory scores of different modified dietary fiber composite steamed breads.

Samples	Completeness	Surface Smoothness	Internal Uniformity	Flexibility	Resilience	Viscosity	Smell	Specific Volume	Totals
WS	9.12 ± 0.94 ^a^	9.54 ± 0.78 ^a^	12.65 ± 1.45 ^a^	8.74 ± 0.61 ^a^	16.23 ± 0.83 ^a^	8.49 ± 0.71 ^c^	11.49 ± 0.57 ^a^	10	86.26 ± 4.76 ^c^
WS-ODF	7.31 ± 1.26 ^d^	6.70 ± 0.83 ^c^	9.60 ± 0.95 ^d^	7.57 ± 0.85 ^c^	11.64 ± 0.62 ^d^	9.28 ± 0.52 ^b^	10.41 ± 0.46 ^b^	7	62.51 ± 5.31 ^c^
WS-HEDF	8.15 ± 1.68 ^c^	7.82 ± 0.64 ^b^	11.47 ± 0.78 ^b^	7.12 ± 0.77 ^d^	14.77 ± 0.75 ^b^	9.13 ± 0.95 ^b^	10.74 ± 0.74 ^b^	10	80.2 ± 6.23 ^c^
WS-UEDF	8.45 ± 1.18 ^b^	7.67 ± 0.76 ^b^	11.66 ± 0.86 ^b^	7.94 ± 0.94 ^b^	15.71 ± 0.97 ^c^	9.45 ± 1.05 ^a^	10.87 ± 1.01 ^b^	10	83.75 ± 5.89 ^c^

Values in the same row with different superscripts are significantly different (*p* < 0.05).

## Data Availability

The original contributions presented in this study are included in the article/Supplementary Materials. Further inquiries can be directed to the corresponding author.

## Supplementary Materials

The following supporting information can be downloaded at: https://www.mdpi.com/article/10.3390/foods14030346/s1, Table S1: The effect of dietary fiber from soybean residue on the thermomechanical Properties of wheat flour. Figure S1: Amplitude scanning.
