# Supplementary material for: Enhancement of Dough Processing and Steamed Bread Quality with Modified Soybean Residue Dietary Fiber
Source: Foods. 2025 Jan 21;14(3):346. doi: 10.3390/foods14030346 (PMC11817071; doi:10.3390/foods14030346)
Supplement: Supplementary file 1 [file foods-14-00346-s001.zip › foods-3397640-supplementary.pdf]

Table S1. The effect of dietary fiber from soybean residue on the thermomechanical Properties of wheat flour

Samples	Water absorption (%)	Dough development time (min)	Dough stability time (min)
WF-dough	61.12±0.21^{f}	3.12±0.08^{e}	6.36±0.45^{c}
ODF-4%	61.64±0.32^{e}	3.37±0.09^{d}	6.78±0.45^{ab}
ODF-6%	62.36±0.35^{d}	3.76±0.10^{b}	6.54±0.78^{b}
ODF-8%	63.53±0.51^{b}	3.89±0.12^{a}	6.51±0.81^{a}
HEDF-4%	62.10±0.43^{d}	3.49±0.11^{c}	6.62±0.78^{b}
HEDF-6%	63.52±0.12^{b}	3.79±0.07^{ab}	6.43±0.90^{c}
HEDF-8%	64.09±0.45^{a}	3.91±0.08^{a}	6.27±0.47^{d}
UEDF-4%	62.81±0.20^{c}	3.52±0.13^{c}	6.88±0.11^{a}
UEDF-6%	63.59±0.49^{b}	3.88±0.09^{a}	6.86±0.23^{a}
UEDF-8%	64.73±0.60^{a}	3.92±0.12^{a}	6.39±0.59^{c}

Values in the same raw with different superscripts are significantly different ($p < 0.05$).

The amplitude scan curve has two regions, including a linear viscoelastic region where G 'and G' are almost parallel, and a nonlinear region where G 'decreases with increasing strain. As shown in Figure S1, all samples have a 1% strain within the linear viscoelastic range.

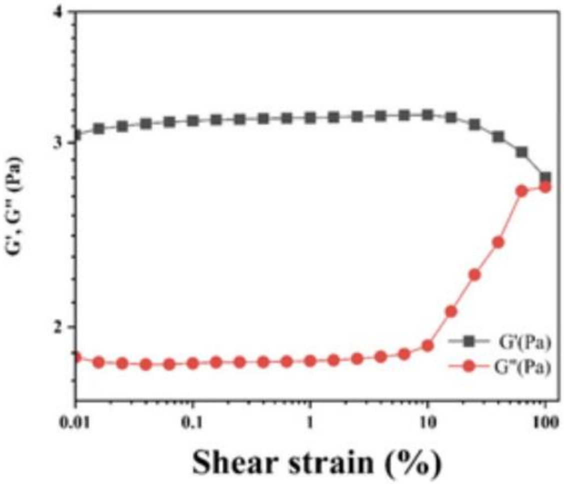

Figure S1. Amplitude scanning.
